# Finding the Optimal Surgical Incision Pattern—A Biomechanical Study

**DOI:** 10.3390/jcm11092600

**Published:** 2022-05-05

**Authors:** Nikolaus Wachtel, Paul I. Heidekrueger, Carolin Brenner, Maximilian Endres, Rainer Burgkart, Carina Micheler, Niklas Thon, Denis Ehrl

**Affiliations:** 1 Division of Hand, Plastic and Aesthetic Surgery, University Hospital, LMU Munich, 81377 Munich, Germany; carolin_brenner@gmx.de (C.B.); s-mendres@helios.med.uni-muenchen.de (M.E.); denis.ehrl@med.uni-muenchen.de (D.E.); 2 Centre of Plastic, Aesthetic, Hand and Reconstructive Surgery, University of Regensburg, 93053 Regensburg, Germany; paul@heidekrueger.net; 3 Department of Orthopaedics and Sports Orthopaedics, University Hospital Rechts der Isar, School of Medicine, Technical University of Munich, 81675 Munich, Germany; rainer.burgkart@tum.de (R.B.); carina.micheler@tum.de (C.M.); 4 Institute for Machine Tools and Industrial Management, School of Engineering and Design, Technical University of Munich, 85748 Garching, Germany; 5 Department of Neurosurgery, Hospital of the University of Munich, 81377 Munich, Germany; niklas.thon@med.uni-muenchen.de

**Keywords:** surgical incision, wound healing, zigzag incision

## Abstract

The closure of wounds and subsequent optimal wound healing is essential to any successful surgical intervention. Especially on parts of the body with limited possibilities for local reconstruction, optimal distribution of load is essential. The aim of the present study was therefore to examine three different incision patterns, conventional straight, Lazy-S and Zigzag, with regard to their biomechanical stability and mode of failure on a porcine skin model. Our results demonstrate the superior biomechanical stability of Lazy-S and Zigzag incision patterns with perpendicular suture placement. This holds true, in particular, for Zigzag incisions, which showed the highest values for all parameters assessed. Moreover, the observed superior stability of Lazy-S and Zigzag incision patterns was diminished when sutures were placed in tensile direction. The conventional straight incision represents the standard access for a large number of surgical procedures. However, we were able to demonstrate the superior biomechanical stability of alternative incision patterns, in particular the Zigzag incision. This is most likely caused by an improved distribution of tensile force across the wound due to the perpendicular placement of sutures. Moreover, this technique offers additional advantages, such as a better overview of the operated area as well as several cosmetic improvements. We therefore advocate that the surgeon should consider the use of a Zigzag incision over a conventional straight incision pattern.

## 1. Introduction

The closure of wounds and subsequent optimal wound healing is one of the fundamental requirements for any successful surgical intervention. A high tensile force across wounds leads to significantly reduced perfusion at the wound margins, resulting in the disruption of collagen synthesis during the wound healing phases [1,2,3,4]. In particular, high tension along an orthogonal axis of the wound causes unstable scars as well as wound dehiscence due to necrosis along the wound margins [1,5]. These complications lead to delayed and/or secondary healing, surgical site infections (SSIs), or even secondary reconstructive surgery [5,6]. An optimal distribution of load and, thus, stability of the wound closure is therefore essential for wound healing. Especially on parts of the body with limited possibilities for local reconstruction, such as the extremities or the scalp.

Regarding optimal tension along the wound margins, several different concepts exist to reduce tension along the suture by carefully choosing the orientation of the incision along predefined lines of reduced tensions [7,8]. Moreover, previous studies demonstrated that the choice of suture material as well as suture type had a significant influence on wound healing [1,9,10,11]. However, little research has gone into whether the type of incision made has an effect on the distribution of the tensile force on the wound and, thus, on wound healing. Our aim was therefore to assess different modes of incisions with regard to their biomechanical stability. We compared the conventional straight, Lazy-S and Zigzag incisions of porcine skin samples with regard to failure load, resistance to deformity and mode of failure.

## 2. Materials and Methods

### 2.1. Porcine Skin Samples

The porcine tissue used for experiments was obtained directly from a local butcher (MRT-Ludwig Leidmann GmbH, Munich, Germany). Pigs were euthanized 4 to 6 months after birth. For experiments, abdominal skin, including Epidermis and Dermis, was removed with a thickness of 5 mm. The final size for skin samples, which were used for testing was 17 × 6 cm. Block randomization was used to determine which skin samples were used in experimental groups and to distribute the skin samples of each animal to the five groups of one experimental series in a balanced manner. For each experimental group, 17 incision-patterns/control skin samples were tested. Thus, a total of 102 individual samples were assessed for their biomechanical stability.

### 2.2. Incisions and Sutures

**Figure 1:**
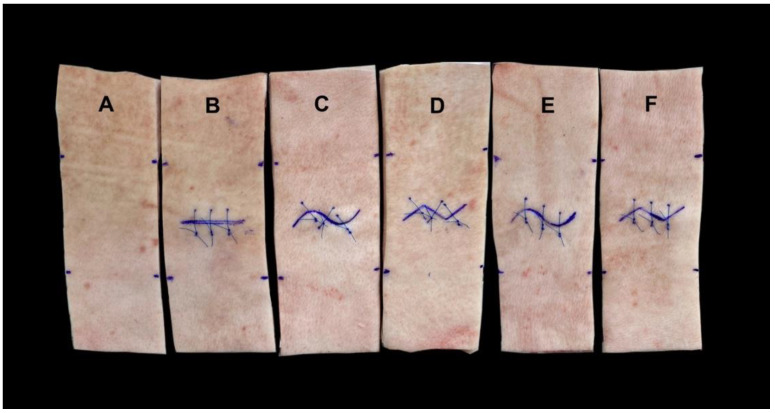
Examples of skin samples used for each experimental group. Control (**A**), conventional straight incision pattern (**B**), Lazy-S (**C**) and Zigzag (**D**) incision patterns with perpendicular (90°) placement of sutures, as well as Lazy-S (**E**) and Zigzag (**F**) incision patterns with placement of sutures in tensile direction were tested for their biomechanical stability.

3D-printed incision-pattern templates were used to standardize skin incisions as well as suture positions (Ultimaker 2+; Ultimaker, Utrecht, The Netherlands). The sutures were placed 8 mm from the edge of the wound and 10 mm apart from each other, as described previously [12]. All incisions used had a length of 4 cm (in a horizontal direction), resulting in 1 cm intact skin bilaterally between the end of the incision and the border of the template (Figure 1).

The angle for both Lazy-S and Zigzag incisions was 112°, giving a complete sinus curve and two prongs for the Zigzag incision for an incision length of 4 cm. Monofilament sutures (Prolene^®^—Ethicon Inc., Bridgewater, NJ, USA) were used as suture material. For all experiments, three standardized single button sutures with four knots each were performed. In order to additionally analyze the effect of suture placement on biomechanical stability of the incision pattern, sutures for Lazy-S and Zigzag incisions were either placed perpendicular (90°) to the incision or parallel to the tensile direction. A skin sample without an incision was used for control.

### 2.3. Biomechanical Testing

For biomechanical testing, porcine skin samples were attached to a tensile testing machine with screw grips (Zwicki 1120, ZwickRoell GmbH & Co. KG, Ulm, Baden-Württemberg, Germany). A preload of 5 N was applied to minimize slack. Sutures were then preconditioned at a velocity of 15 mm/min to be continuously elongated until failure at a velocity of 100 mm/min. The tensile load test outputs were plotted on a load-deformation curve (TestXpert V12.0 ZwickRoell GmbH & Co. KG, Ulm, Baden-Württemberg, Germany). A custom written MATLAB code (MATLAB R2020a, The MathWorks, Inc., Natick, MA, USA) was used to determine resistance to deformity (stiffness), first suture rupture load (i.e., first failure load) as well as second and third suture rupture load (i.e., final failure load). The resistance to deformity of the incision patterns was determined within the linear elastic area of the load-deformation curve. The preconditioning data were removed and by means of the coefficient of determination (R^2^), the linear elastic area was identified to calculate resistance to deformity [13]. Means and standard deviations were calculated for the different groups. Tensile testing was filmed using a Legria HF M31 video camera (Canon Co., Ltd., Ohta-ku, Tokyo, Japan) to document the mode of failure (pull-out vs. suture breakage) [14]. Considering the cost for acquisition and maintenance of the biomechanical testing equipment as well study-specific materials (skin samples, suture material, 3D-printed incision-pattern templates) the average cost per skin sample tested was approximately EUR 4.80.

### 2.4. Standardization and Statistical Analysis

We experienced considerable variations in preliminary experiments for biomechanical properties of control skin samples from different donor animals. We therefore used block-randomization of samples and standardized measurement to be expressed as mean ratios with standard deviation (SD) as compared to controls. As control samples partially loosened from the clamp before tissue rupture (at a force of least 400 N) we could not use the ultimate failure load or elastic limit of controls as reference force. Instead, a standardization model, using the linear elastic range of each control was implemented. Thus, a fixed force value of 300 N, which was well within the linear-elastic range of all control samples, was defined. The corresponding displacement of the control skin sample for this fixed value was used as a reference baseline. Subsequently, the force value needed for 10% of additional displacement was obtained for each control. This value was then used as a standardized reference, taking the individual elastic properties of the different donor animals into account.

One-way analysis of variance (ANOVA), followed by the Tukey–Kramer test for multiple comparisons was conducted to assess effects of incision patterns techniques on resistance to deformity and first suture failure load (first failure load) as well as second and third suture failure load. The Student’s t-test was used to test for significant differences between suture placement (perpendicular vs. tensile direction) for Lazy-S and Zigzag incision patterns. A *p*-value of <0.05 was considered statistically significant. GraphPad Prism 6 (GraphPad Software, Inc., San Diego, CA, USA) was used as software for statistical analysis.

## 3. Results

**Figure 2:**
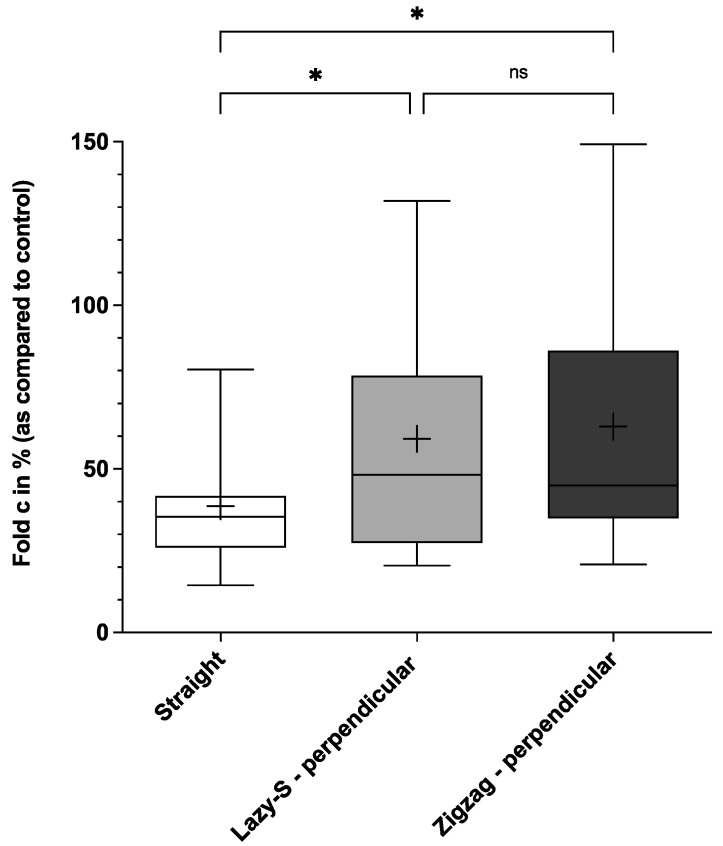
Comparison of resistance to deformity (c) between three main incision patterns. Conventional straight, Lazy-S and Zigzag incisions (both with perpendicular (90°) placement of sutures) were tested for their biomechanical stability. The mean is indicated by a cross. The bottom and top of the box are the 25th and 75th percentile. Error bars (whiskers) indicate the 10th and 90th percentile. * *p* < 0.05; ns, not significant. For each group 17 incision-patterns were tested.

This Lazy-S, as well as Zigzag incisions with perpendicular suture placement, had a significantly higher resistance to deformity when compared to conventional straight incisions (mean of 59.2 (43.0)% and 63.0 (42.7)% vs. 38.7 (20.7)%; both *p* < 0.05) (Figure 2).

For both Lazy-S and Zigzag incision patterns resistance to deformity was reduced when sutures were placed in tensile direction. As compared to orientation of sutures in a perpendicular manner, the mean resistance to deformity was 36.7 (20.5)% vs. 59.2 (43.0)% for Lazy-S and 44.9 (25.6)% vs. 63.0 (42.7)% for Zigzag incisions. The difference was statistically significant for Lazy-S incision patterns (*p* < 0.05). Consequently, no statistically significant difference in resistance to deformity was observed for both Lazy-S and Zigzag incision patterns with suture placement in tensile direction when compared to straight incision patterns (data not shown; also refer to Supplementary Data).

**Figure 3:**
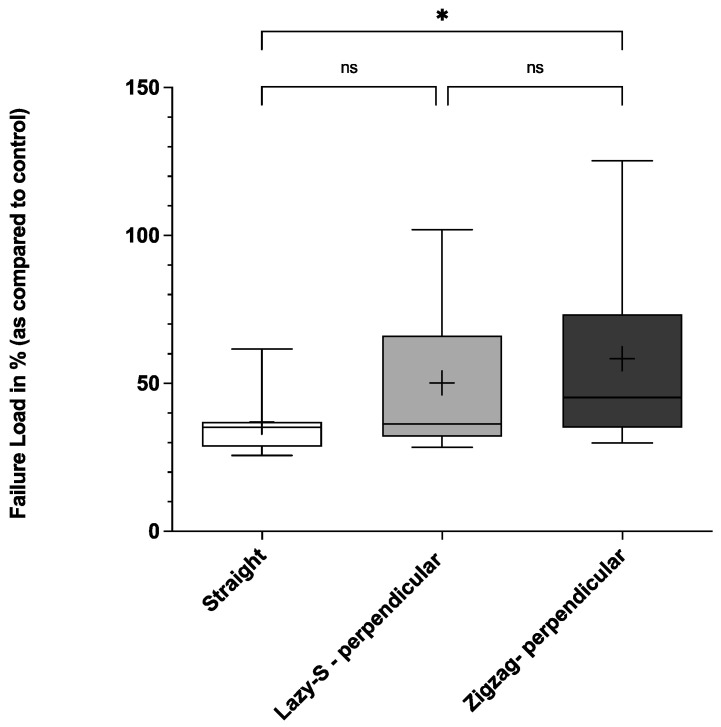
First suture failure load (first failure load) for three main incision patterns. Conventional straight, Lazy-S and Zigzag incisions (both with perpendicular (90°) placement of sutures) were tested for their biomechanical stability. The mean is indicated by a cross. The bottom and top of the box are the 25th and 75th percentile. Error bars (whiskers) indicate the 10th and 90th percentile. * *p* < 0.05; ns, not significant. For each group 17 incision-patterns were tested.

**Figure 4:**
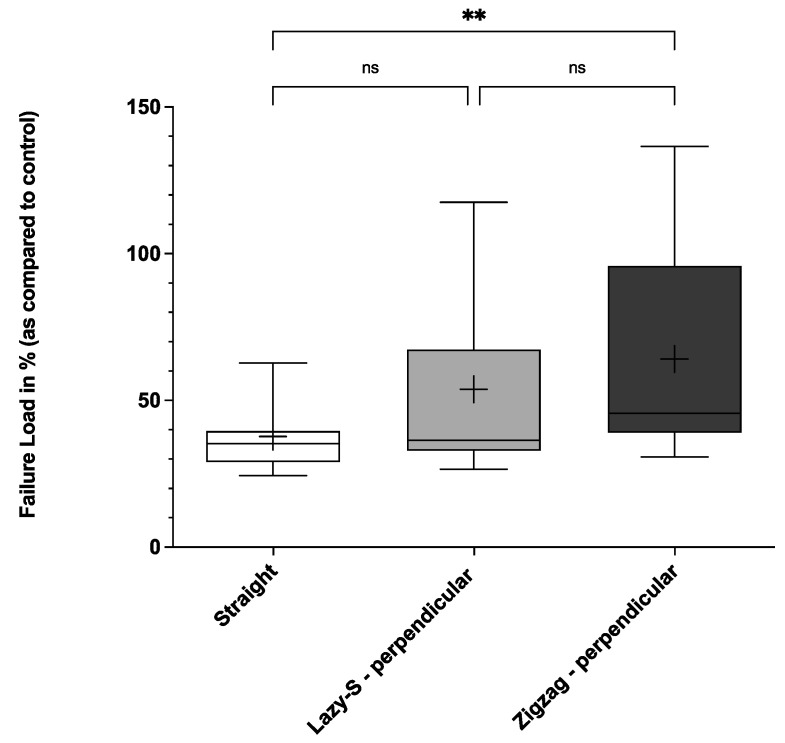
Second suture failure load for three main incision patterns. Conventional straight, Lazy-S and Zigzag incisions (both with perpendicular (90°) placement of sutures) were tested for their biomechanical stability. The mean is indicated by a cross. The bottom and top of the box are the 25th and 75th percentile. Error bars (whiskers) indicate the 10th and 90th percentile. ** *p* < 0.01; ns, not significant. For each group 17 incision-patterns were tested.

**Figure 5:**
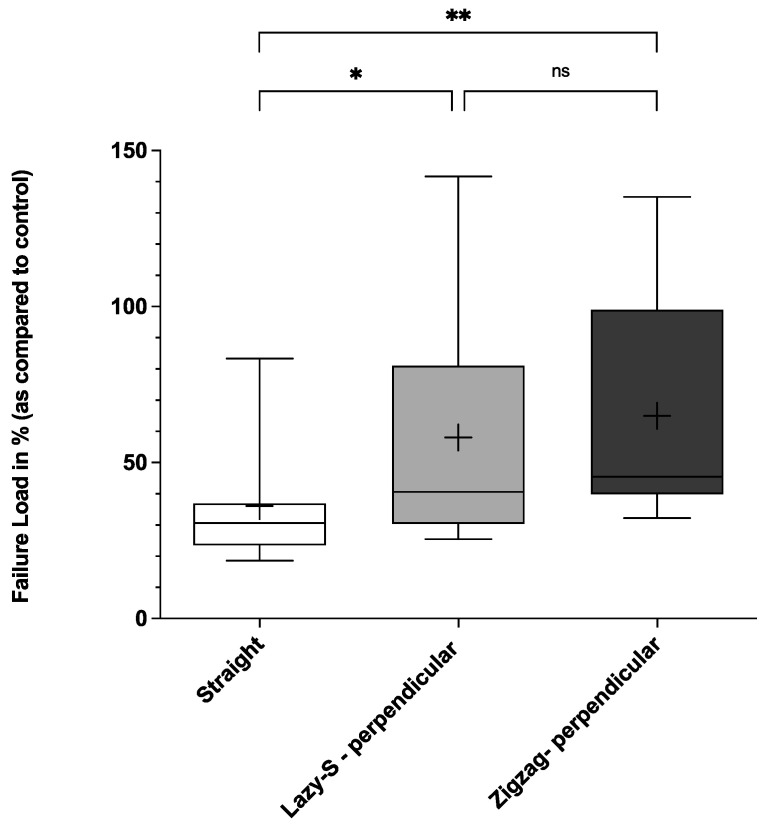
Third suture failure load (final failure load) for three main incision patterns. Conventional straight, Lazy-S and Zigzag incisions (both with perpendicular (90°) placement of sutures) were tested for their biomechanical stability. The mean is indicated by a cross. The bottom and top of the box are the 25th and 75th percentile. Error bars (whiskers) indicate the 10th and 90th percentile. ** *p* < 0.01; * *p* < 0.05; ns, not significant. For each group 17 incision-patterns were tested.

When analyzing the force needed leading to suture rupture, Zigzag incisions with perpendicular suture placement demonstrated highest biomechanical stability (Figure 3, Figure 4 and Figure 5).

Thus, mean failure load for first, second and third sutures was significantly higher for Zigzag incisions when compared to conventional straight incisions (58.3 (36.1)%, 64.0 (38.7)%, and 65.0 (39.4)% vs. 36.9 (15.4)%, 37.7 (15.1)% and 36.1 (20.7)%, respectively; *p* < 0.05). Lazy-S incision patterns also showed higher stability when compared to straight incision patterns. However, only for the third suture failure load (final failure load) was this difference statistically significant (58.0 (45.2)% vs. 36.1 (20.7)%; *p* < 0.05) (Figure 5).

Similar to the results for resistance to deformity, suture placement in tensile direction leads to lower load bearing capacities of sutures for both Lazy-S and Zigzag incisions. This difference was significant for the third suture failure load (ultimate failure load) of Lazy-S incision patterns (58.0 (45.2)% vs. 32.7 (11.9)%; *p* < 0.05). Consequently, no difference in force necessary for suture rupture was observed for both Lazy-S and Zigzag incision patterns with suture placement in tensile direction when compared to straight incision patterns (data not shown; also refer to Supplementary Data).

With regard to mode of failure, all sutures failed by suture breakage. Moreover, no clear sequence of suture breakage was observed.

## 4. Discussion

This study demonstrates the superior biomechanical stability of Zigzag and Lazy-S incision patterns with perpendicular suture placement over a conventional straight incision. This holds true in particular for Zigzag incisions, which showed significantly higher values for all parameters assessed (Figure 2, Figure 3, Figure 4 and Figure 5). Our results are therefore in concordance with previous clinical studies that observed the superiority of Zigzag incisions over conventional straight incisions [15,16,17]. While these predominantly show an advantage in patient satisfaction and cosmetic aspects, the current study demonstrates the superior biomechanical stability of a Zigzag incision pattern.

A likely reason for the increased stability we observed for Zigzag and Lazy-S incisions is the placement of sutures not in tensile direction but in a perpendicular orientation towards the incision. This may allow for a better distribution of force when under stress: due to their oblique arrangement, application of force initially leads to partial alignment of sutures in the direction of the pull. This leads to additional deformation of the skin and subsequently to minimal parallel displacement of wound margins against each other. The overall result is a lower force that is being transmitted through the threads while the tensile force applied through the machine remains the same. This hypothesis would also explain why suture placement in tensile direction for Zigzag and Lazy-S incision patterns reduced stability to a level similar to the one observed for conventional straight incisions. Indeed, a similar mechanism was recently proposed for different tenorrhaphy techniques. Here, the authors argue that oblique cross-stitches permit a better distribution of force, and therefore a higher stability, when compared to regular mattress sutures [15,18]. Moreover, the Zigzag incision pattern seems to be better suited for this mechanism of force distribution than Lazy-S incisions as we observed superior biomechanical properties for this group.

With regard to previous articles assessing tension along wound margins and healing, several decades ago Borges et al. observed the natural ridges and furrows caused by the direction of muscle pull and its effect on the overlying skin [19]. It was concluded that placement of incisions along these predefined lines, termed relaxed skin tension lines (RSTL), results in better functional as well as cosmetic results. However, in 2018 Paul et al. revised these assumptions and presented the concept of biodynamic excisional skin (BEST) lines for an optimal orientation of incisions at the extremities, the trunk, and the scalp [7,8]. In a large clinical study, the authors were able to correct the initial assumptions of RSTL by Borges and colleagues with regard to optimal wound closure by demonstrating that placement of skin excisions along the established BEST lines lead to significantly lower tension across the wound margins in vivo. Therefore, the orientation of incisions should primarily follow the BEST lines, except for surgery in the face. Here, incisions along the RSTL are most likely to result in ideal wound healing.

**Figure 6:**
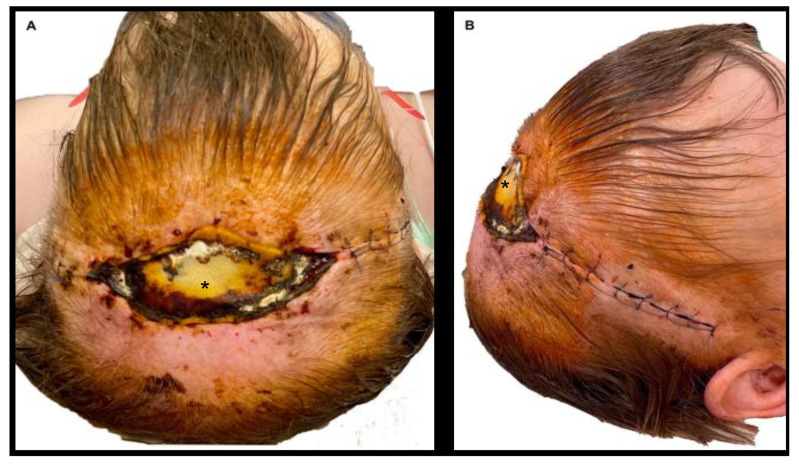
(**A,B**) Example of a patient with SSI and disrupted wound healing after coronal craniotomy with a straight incision due to recurrent meningioma. The asterisk marks a temporary wound dressing (EpiGARD^®^; Biovision GmbH, Ilmenau, Germany). The patient needed free flap tissue coverage in the course of therapy.

The current study complements these conclusions; with regard to biomechanical stability and optimal wound healing, the surgeon should consider using a Zigzag (and with lesser effect a Lazy-S) incision with perpendicular suture placement, orientated along the BEST lines or RSTL for the face. Following these guidelines will significantly reduce tension along the orthogonal axis of the wound and, thus, is likely to reduce complications such as delayed secondary healing or SSIs. This holds true especially in areas with minor soft tissue coverage and limited local reconstructive options such as the head and over large joints of the extremities. Here, a suture with optimal load distribution seems crucial as delayed secondary healing is often not possible (Figure 6). Additionally, a Zigzag incision offers the significant advantage of increasing exposure, thereby improving patient safety by facilitating the surgical procedure [20].

From a cosmetic standpoint, a Zigzag or a Lazy-S incision may be more noticeable at first due to their shape as well as overall incision length. However, in general the result tends to be superior after complete wound healing when compared to conventional straight incisions. This is due to a reduced scar width, possibly the result of a better distribution of tensile force across the incision [20]. Moreover, on hair-bearing skin, in particular the scalp, a Zigzag incision pattern results in considerably less visible scaring due to a natural separation of the hair, thereby significantly improving the cosmetic result and patient satisfaction. Therefore, a Zigzag modification of the conventional coronal incision for frontotemporal craniotomy has been proposed by several authors in the past [15,16,17]. For the face, the orientation of the 14 aesthetic units and their subunits must be additionally taken into account [21,22]. If possible, the incision should not cross from one unit to the next, thereby preserving the aesthetic appearance and symmetry of the face.

A possible disadvantage of the Zigzag as well as Lazy-S technique is the potential risk of necrosis of the flap tips [20]. Therefore, in the current study, a large angle of 112° was chosen for these incision patterns, resulting in a low length to width ratio of 1 to 3 that is unlikely to impair vital blood supply to the distal parts of the flap. Thus, numerous successful random pattern flap techniques have been previously described with a safe flap viability with a considerable larger length to width ratio of at least 2 to 1 [23,24]. Similarly, Z Plasty transposition flaps are successfully performed with much lower angles than the one used in this study; the classic Z Plasty is described with an angle of 60° [25,26]. Moreover, lower angles of 45° and less have been described in the past [27,28]. Necrosis of flap tips for the incision patterns described in the current study is therefore unlikely.

In this study we established an in vitro model to objectively assess the stability of skin samples with or without various incision patterns. Similar models (including endpoints and clinimetrics) have been successfully used in the past, in particular, to assess the stability of tendon sutures and tenorrhaphies [14,29,30]. However, the findings of our study are significantly limited by its (in vitro) set-up. While porcine skin models show many similarities to human skin, it is unclear whether findings are completely transferable to biomechanical properties of human skin [31,32]. Additionally, by using a cadaver model, we are not able to draw any deductions on effects mitigated by normal tissue biology or assess changes in suture stability during the course of wound healing. Currently, we are therefore conducting a prospective clinical study that assesses wound healing and general outcome in different craniotomy incision patterns to further validate the findings of this study.

We used 3D-printed incision-pattern templates to standardize skin incisions as well as suture positions and all sutures had the same sequence as well as number of knots. However, the force used to tighten knots was not standardized in this study. While all sutures failed by direct suture breakage, a varying force to tighten knots may still have influenced our findings. Finally, the considerable variations of biomechanical properties of skin samples from different donor animals further limit the conclusions that can be drawn from this study. While we were able to partially account for individual diversity by using block-randomization as well as standardization of experimental groups, these approaches are unlikely to factor in the whole spectrum of individual diversity.

Nevertheless, our results demonstrate the biomechanical superiority of Zigzag and (to a lesser extent) Lazy-S incision patterns with perpendicular suture placement over a conventional straight skin incision. A likely hypothesis for the observed effect is the different arrangement of sutures in relation to the tensile direction. With regard to additional advantages, which have been described by previous studies (e.g., improved exposure and aesthetic outcome), the Zigzag incision pattern in particular should be further assessed as a possible standard incision for surgical procedures. Being aware of the above-mentioned limitations, we advocate for subsequent studies that assess the Zigzag incision pattern in an in vivo set-up.

## Data Availability

Not applicable.

## Supplementary Materials

The following supporting information can be downloaded at: https://www.mdpi.com/article/10.3390/jcm11092600/s1, Table S1. Mean relative values for experimental groups. Data is expressed in % (SD) as compared to controls. Different superscripts (i.e., *a* and *b*) indicate statistically significant differences among incision patterns at p of at least < 0.05 (the superscript *ab* indicates no significant difference when compared to other incision patterns). For each experimental group 17 incision-patterns were tested.
