# Supplementary material for: Finding the Optimal Surgical Incision Pattern—A Biomechanical Study
Source: J Clin Med. 2022 May 5;11(9):2600. doi: 10.3390/jcm11092600 (PMC9099478; doi:10.3390/jcm11092600)
Supplement: Supplementary file 1 [file jcm-11-02600-s001.zip › jcm-1656479-supplementary.pdf]

Supplementary Data

	Straight	Lazy-S - perpendicular	Zigzag - perpendicular	Lazy-S - tensile direction	Zigzag - tensile direction
**Resistance to Deformity** ***(%)***	38.7 (20.7)^{a}	59.2 (43.0)^{b}	63.0 (42.7)^{b}	36.7 (20.5)^{a}	44.9 (25.6)^{ab}
**First suture failure load (first failure load)** ***(%)***	36.9 (15.4)^{a}	50.2 (28.3)^{a}	58.3 (36.1)^{b}	35.7 (8.9)^{ab}	41.7 (14.4)^{ab}
**Second suture failure load** ***(%)***	37.7 (15.1)^{a}	53.8 (37.5)^{a}	64.0 (38.7)^{b}	35.4 (10.3)^{ab}	43.4 (18.6)^{ab}
**Third suture failure load (final failure load) (%)**	36.1 (20.7)^{a}	58.0 (45.2)^{b}	65.0 (39.4)^{b}	32.7 (11.9)^{a}	43.7 (24.5)^{ab}

Table S1. Mean relative values for experimental groups. Data is expressed in % (SD) as compared to controls. Different superscripts indicate statistically significant differences among groups at p of at least < 0.05. For each experimental group, 17 incision-patterns were tested.
